# Ovariotomy

**Published:** 1867-06

**Authors:** S. D. Jackson

**Affiliations:** Chicago, Ill.


### CHICAGO MEDICAL JOURNAL.

Vol. XXIV. JUNE, 1867. No. 6.

### Original Contributions.

### OVARIOTOMY.

By S. D. Jackson, M.D., Chicago, Ill.

Mrs. R., a native of Denmark, aged 28, married, states that when 13 years of age she had a severe attack of rheumatic fever from which she gradually recovered; at the age of 19 her menses first made their appearance but have subsequently been quite irregular. Three years ago, she was subject to hemoptyses. Within the past three years she has given birth to two children,—the latter being born in August, 1866,—and notwithstanding that gestation as well as delivery was perfectly normal, yet both children died in less than a month after birth.

Shortly after the last confinement she became subject to more or less constant and severe pains about the right iliac region and loins, the pains extending down the right thigh; she describes these pains as of a "bearing down" nature. When I first saw her, which was in December, 1866, her general health was very much impaired so she could perform her domestic duties only with great difficulty on account of the pains and weakness. My colleague, Dr. Larron, saw her with me at this time and coincided in the opinion that the difficulty was one of ovarian dropsy; the dulness in the right iliac region was circumscribed and retained the location irrespective of the patient's position. Examination per vaginam revealed descent of the womb with considerable retroversion. There was no protrusion of the walls of the vagina such as will be found in ascites; the os uteri was enlarged and its surface being excoriated with considerable ropy discharge from the mouth of the os. The uterine sound was easily introduced with its point turned backwards and, by everting it, the fundus would be raised to its normal position, but on withdrawing the sound it would immediately resume its former situation. Although skeptical as to its beneficial result, I ordered diuretics consisting of infusion of digitalis and nitrate of potash internally, with tincture of iodine locally, and free use of milk as nourishment. She was ordered to stay in bed, use cold hip baths and cold injections into the vagina. She was also ordered a mixture of sulphate of magnesia and iron. As she at this time lived 16 miles in the country, I did not tend her regularly and saw her again first in the latter part of February, when she moved to this city, at which time the dropsical effusion was increased to considerable extent, so that she was confined to her bed nearly all the time. Her urgent desire was that something might be done in order to relieve her distress, which was daily becoming more and more alarming. I frankly told her the *modus operandi* which in my opinion offered her any chance of relief, namely, paracentesis and ovariotomy, the former as one that was of but little danger and would give her temporary relief; and the latter as very formidable (perhaps exaggerating the danger somewhat) but if successful would promise a permanent cure. She herself, as well as her husband, unhesitatingly preferred ovariotomy, and requested that I would operate as soon as convenient. My colleague, Dr. Larron, fully concurred with me in these views. I, therefore, in order to prepare her for the operation, ordered liberal diet with tonics, and fixed on the 28th of March as the day for the operation, this being just a fortnight after her last menstruation. Meanwhile the effusion was constantly increasing, and on the morning of the 28th of March the abdomen presented the following measurements: circumference about the umbilicus, 40 inches; distance between ensiform process and symphyies pubis, 21 inches; and between the umbilicus and pubes, 10 inches.

The bladder and rectum evacuated she was chloroformed, and when partially under the influence removed to an adjoining room, the temperature of which was 85° F., and placed on the operating table, and when fully anæsthetized one fourth gr. morph. sulph. was administered by subcutaneous injection, in order to prolong the effect of the chloroform. At forty minutes past 10 o'clock A.M., I proceeded to operate, assisted by Drs. Larron, Paoli, and Quales. An incision 4 inches long was made in the linea alba, commencing about four inches below the umbilicus and extending to within about 3 inches of the symphysis pubis. The various layers of the abdominal wall (which was very thin) were now carefully divided. When the hemorrhage, which was very slight, was controlled the peritoneal lining was divided and the cyst presented itself in the opening. In accordance with late English authors I did not attempt to remove the cyst as a whole, but introduced Spencer Mill's trocar through its walls and withdrew the greater portion of its contents, which consisted of a limpid serous fluid of a greenish color; the cyst, now collapsed, was seized by a pair of forceps, its adhesions to the abdominal wall anteriorly broken up, and by gentle manipulations it was forced out through the opening, when its pedunculated extremity was found attached to the right ovary and adjacent broad ligament. The peduncle was rather short and flattened, being about 2 inches broad. The attachment to the broad ligament was about one inch from the adjacent part of the uterus, this organ as well as the adjacent parts being perfectly healthy. The parallel clamp was now applied over the peduncle as near the cyst as possible on account of the shortness of the peduncle. The clamp was screwed sufficiently tight and the cyst removed; (the time elapsed since the beginning of the operation was 30 m.) There was some oozing of blood at the seat of the adhesions which was controlled by persulphate of iron, torsion, and ice. All coagulas of blood and other foreign substances in the peritoneal cavity were now scrupulously removed, when the wound was closed by four interrupted sutures penetrating through the entire thickness of the abdominal wall; between these three more superficial sutures were applied. The wound was covered with a piece of oiled linen—and the whole of the abdomen painted over with collodion. On section, the peduncle was found to contain a large number of large vessels, but the bleeding was controlled perfectly by the clamps. The divided extremity of the peduncle was saturated with the muriated tincture of iron. The abdomen was covered with several layers of wadding and a broad bandage applied round the whole, and the patient carried back to bed, having remained on the operating table about two hours. The fluid contained in the cyst was not less than 8 gallons; the weight of cyst and contents was 66 pounds. She was ordered port wine *ad libitum* and tincture opium sufficient to control the pains. The pulse was rather weak and the frequency about 100 per minute during the operation; on the evening of the 28th the pulse was 104 per minute, and she complained of some pain in the loins and a desire to urinate without being able to evacuate the contents of the bladder, which had to be drawn off by means of a catheter. She was ordered gtt. xx of laudanum every 6 hours.

*March 29th.* Slept several hours during the night and feels somewhat stronger. Pulse 108 per minute; skin moist and warm; intense thirst. Bladder catheterized; treatment continued.

*March 30th.* Slept pretty well during the night. Pulse 108 per minute; pain about the abdomen diminished; coughs some, and was ordered an expectorant.

*March 31st.* Slept well; pulse 98 per minute; wound united down to the peduncle, slough of the latter black and dry; no signs of peritonitis; sutures removed; liberal diet and port wine *ad libitum;* bladder evacuated by means of catheter, the urine contained considerable mucus.

*April 1st.* Pulse 100 per minute; coughs considerably, feels otherwise comfortable; appetite good.

*April 2d.* Pulse 104 per minute; cough less troublesome. Injection of cold water into the rectum; no evacuation since the operation was performed. Ordered decoction of cinchona and senega, teaspoonful every 4 hours.

*April 2d.* Spontaneous evacuation from the bowels and bladder; pulse 104 per minute, no tympanites; edges of the wound slightly separated, but only the integument; some swelling and tenderness and pressure about the right illiac region; a slight fœtid discharge about the peduncle.

*April 4th.* Pulse 116; no sleep; cough troublesome. Was ordered the following: decoc. cinch. infus. herb digitalis āā ℥iij, potassæ nitrat. and succo liqueret āā ʒij m&s. Teaspoonful every 2 hours.

*April 5th.* Rested well during the night, and feels comfortable. Pulse 96 per minute. Clamps removed, peduncle adhered to the edges of the wound.

*April 7th.* Pulse 90 per minute; complains of fever occurring about noon. Ordered quinia sulph gr. v, twice a day.

*April 9th.* No fever. Pulse 72 per minute, and on the whole apparently comfortable. The circumference about the umbilicus 27 inches; distance between ensiform process and symphysis pubis 11½ inches, and between the umbilicus and symphysis pubis 5½ inches.

*April 15th.* Wound entirely closed; permitted to leave the bed.

*April 21st.* Complains of transient pains about the loins.

*April 22d.* Gradually improving; evidence of approaching menstruation; situation of uterus perfectly normal. Pulse 72 per minute.

*April 25th.* Walks out and must be considered fully recovered; wound entirely healed; no abnormal discharges from the vagina.

