# Erratum

**Published:** 1867-09

**Authors:** 


### *Erratum.*

Our attention has been called to a typographical error in the June No. of the JOURNAL. The article on "Ovariotomy" was contributed by S. D. Jacobson, M.D., and not *Jackson*, as the types made it.

